# Genetic Modification of CD8^+^ T Cells to Express EGFR: Potential Application for Adoptive T Cell Therapies

**DOI:** 10.3389/fimmu.2019.02990

**Published:** 2019-12-20

**Authors:** Teresa Lozano, Silvia Chocarro, Celia Martin, Aritz Lasarte-Cia, Cynthia del Valle, Marta Gorraiz, Patricia Sarrión, Marina Ruiz de Galarreta, Amaia Lujambio, Sandra Hervás-Stubbs, Pablo Sarobe, Noelia Casares, Juan J. Lasarte

**Affiliations:** ^1^Immunology and Immunotherapy Program, Center for Applied Medical Research, University of Navarra, Pamplona, Spain; ^2^Department of Oncological Sciences, Icahn School of Medicine at Mount Sinai, New York, NY, United States

**Keywords:** epidermal growth factor receptor EGFR, EGFR ligands, adoptive cell therapy, genetic modification, CD8^+^ T cells, tumor microenvironment, hepatocellular carcinoma

## Abstract

Adoptive immunotherapy with *ex vivo*-expanded tumor-infiltrating lymphocytes (TILs) has achieved objective clinical responses in a significant number of patients with cancer. The failure of many patients to develop long-term tumor control may be, in part, due to exhaustion of transferred T cells in the presence of a hostile tumor microenvironment. In several tumor types, growth and survival of carcinoma cells appear to be sustained by a network of receptors/ligands of the ErbB family. We speculated that if transferred T cells could benefit from EGFR ligands produced by the tumor, they might proliferate better and exert their anti-tumor activities more efficiently. We found that CD8^+^ T cells transduced with a retrovirus to express EGFR responded to EGFR ligands activating the EGFR signaling pathway. These EGFR-expressing effector T cells proliferated better and produced more IFN-γ and TNF-α in the presence of EGFR ligands produced by tumor cells *in vitro*. EGFR-expressing CD8 T cells from OT-1 mice were more efficient killing B16-OVA cells than control OT-1 CD8 T cells. Importantly, EGFR-expressing OT-1 T cells injected into B16-OVA tumor bearing mice were recruited into the tumor, expressed lower levels of the exhaustion markers PD1, TIGIT, and LAG3, and were more efficient in delaying tumor growth. Our results suggest that genetic modification of CD8^+^ T cells to express EGFR might be considered in immunotherapeutic strategies based on adoptive transfer of anti-tumor T cells against cancers expressing EGFR ligands.

## Introduction

Adoptive cell therapy (ACT) with *ex vivo-*expanded tumor-infiltrating lymphocytes (TILs) has achieved objective clinical responses in a significant fraction of patients with cancer (1). The success of ACT is largely related to their capacity to infiltrate the tumor, persist in the host, and exert their anti-tumoral activity after their transfer. The failure of many patients to develop long-term tumor control may be, in part, due to exhaustion and apoptosis of the transferred T cells. The i*n vivo* persistence of transferred T cells can vary from hours or days to weeks (2). T cell persistence can depend largely on the manner in which T cells were expanded *in vitro*, the conditions of cell administration, and importantly, on the tumor microenvironment.

The tumor microenvironment is a hostile medium with an abundance of inhibitory factors for lymphocytes. However, tumor microenvironment may contain factors that can favor cell proliferation. This fact prompted several investigations aimed at assembling T cells with cytokine receptors such as CSF-1 (3) or inverted cytokine receptors IL-4Ralpha ectodomain/IL-2 beta(c) endodomain (4), IL4/IL7 (5), or IL4/IL21 (6) among others, to respond positively to ligands expressed into the tumor. In this work, we focussed on the epidermal growth factor receptor (EGFR, also known as ErbB1 or HER-1) and their ligands. Indeed, their overexpression confer a proliferative advantage to tumor cells and have been correlated with progression to invasion and metastasis in a wide variety of cancers, including esophageal (7), gastric (8), hepatocellular (9, 10), head and neck (11) lung (12), or colorectal carcinomas (13, 14). Thus, in addition to overexpression of the EGF receptor, its ligands epidermal growth factor (EGF), transforming growth factor-α TGF-α, amphiregulin (AR), betacellulin (BTC), heparin-binding EGF-like growth factor (HB-EGF), and epiregulin (EREG) have been reported to be upregulated in tumor tissues (9–15). For these reasons, EGFR and its downstream signaling molecules are considered as targets for therapeutic interventions in cancer (16).

The ErbB ligands are synthesized as membrane-anchored precursors (17) that are released as active factors from the cell surface by transmembrane proteases of the “disintegrin and metalloproteinases” (ADAM) type (18, 19). The soluble growth factors may bind their cognate receptors in an autocrine or paracrine manner, although membrane anchored ErbB ligands can also signal to adjacent cells in a juxtacrine fashion (17). These auto/justa/paracrine loops lead to signaling via the tyrosine kinase domain and may provide an advantage for tumor development and metastatic progression.

EGFR is well-established to be ubiquitously expressed but, in general, is thought to be absent in the hematopoietic cell linage with the exception of sporadic expression described in monocytes (20) or plasma cells (21). Recent reports have also shown that Foxp3+ regulatory T (Treg) cells express EGFR under inflammatory conditions. The EGFR ligand amphiregulin (AR) markedly enhances Treg cell function *in vitro* and *in vivo* (22–24). These findings reveal EGFR as a component in the regulation of local immune responses as well as in tissue protection after damage (22, 25). Curiously, conventional effector T cells do not express EGFR (22) and thus, they do not benefit from the presence of EGFR ligands in the tumor microenvironment. A recent report has shown that, under certain circumstances Th2 cells can express EGFR allowing them to produce inflammatory cytokines that may protect the host from infections (26). We speculated that if adoptively transferred T cells could respond to these ligands they might be able to persist and proliferate better within the tumor microenvironment. We modified genetically anti-tumor CD8^+^ T cells to express EGFR and studied the effect of EGFR ligands on their function *in vitro* and *in vivo*.

## Materials and Methods

### Cell Lines and Mice

EL-4, CT26, B16F10, B16-OVA, E.G7.OVA, 4T1, A20, MC38 (ATCC, American Type Culture Collection), Hepa129 (provided by Dr. M Gonzalez-Carmona, Bonn, Germany), 5TGM1 (kindly provided by Dr. Oyajobi, TX), PM-299L (provided by Dr. Lujambio, NY) cell lines were cultured in complete medium (RPMI 1640 or DMEM containing 10% FCS, antibiotics, 2 mM glutamine and 50 μM 2-ME. The Platinum Ecotropic cell line (Plat-E, ATCC) was cultured in DMEM supplemented with 10% FCS and the selection antibiotics puromycin (100 ug/ml) and blasticidin (10 ug/ml).

Female C57BL/6 mice were purchased from Harlan Laboratories. OT-1 transgenic mice (C57BL/6-Tg [Tcra/Tcrb] 1100 Mjb/J) with a TCR recognizing H2-Kb-restricted OVA (257-264 SIINFEKL peptide) and CD45.1 transgenic C57BL/6 mice (B6.SJL-PtprcaPep3b/BoyJ mice) were obtained from Jackson Laboratory (Bar Harbor, ME). OT-1 mice were crossed with CD45.1 mice at our animal facility to obtain homozygous OT-1 × CD45.1 mice.

### RNA Isolation, Quantitative Real-Time PCR (qPCR)

Total RNA was isolated from different tumor cell lines (5 × 10^6^ cells) and from murine tumor biopsies (homogenized in 1 mL of Ultraspec solution, Biotex, Houston, TX) using MagMax 96 total kit (Ambion). After DNAse treatment (Invitrogen) and retrotranscription (Invitrogen), the expression of target genes was measured by a real-time PCR reaction using specific primers (Table 1) and the iQ SYBR Green Supermix (Bio-Rad, Hercules, CA). β-Actin was used to normalize gene expression. mRNA values were represented by the formula: 2^ΔCt^, where Δ*C*_t_. indicates the difference in the threshold cycle between β-Actin and target genes.

**Table 1:** Primers used for iQ-PCR.

**Gene**	**Sense primer (5^**′**^-3^**′**^)**	**Antisense primer (5^**′**^-3^**′**^)**
Ar	CTGCTGGTCTTAGGCTCAGG	CCAGGTTCTCGATGTATCTGC
Btc	CAAGCATTACTGCATCCATG	GGTCTCTTGAATATCTTCAC
EGF	CCCTGGATCCTATTACTGCAC	GAAAGCAATCACATTCCCAGG
EGFR	CTTCTTAAAGACCATCCAGG	TTTCTGGCAGTTCTCCTCTC
EPGN	CTACATAGAAGAACCTGTAGC	TAGCAATAGAAGACAGCAAG
EREG	ACAAAGTGTAGCTCTGACATG	CGATTTCTGTACCATCTGCAG
HB-EGF	ATGAAGCTGCTGCCGTCGGTG	TGGATGCAGTAGTCCTTGTATTTC
TGF-α	GCCCAGATTCCCACACTCAG	AGGACAGCCAGGGCCAC

*Ar, amphiregulin; Btc, betacellulin; EGF, epidermal growth factor; EGFR, EGF receptor; EPGN, epithelian mitogen; EREG, epiregulin; HB-EGF, Heparin-binding EGF-like growth factor; TGF-a, Transforming growth factor alpha*.

### Measurement of EGF Produced by Tumor Cell Lines

EGF present into the tumor microenvironment was measured in tumor cell extracts obtained from mice bearing B16-OVA, PM299L, or Hepa129 derived tumors using the DuoSet Mouse EGF ELISA kit (R&D, MN, USA) according to manufacturer's instructions.

### Retrovirus Production and Lymphocyte Transduction

The retrovirus expression plasmid KMV IRES-GFP was kindly provided by Dr. Rao (LIAI; La Jolla, California). Plasmids expressing EGFR were prepared by Genscript (NJ, USA) and used to subclone them into the KMV IRES EGFR-GFP plasmid. The Plat-E cell line as used for retrovirus production. Packaging cells were transfected with 5 μg of retroviral plasmid (KMV-IRES-GFP or KMV-IRES-EGFR-GFP) along with 2.5 μg pCL-Eco plasmid DNA using lipofectamine (ThermoFisher Scientific, MA, USA. Retroviral supernatants were collected at 48 and 72 h. CD8^+^ T cells were isolated from the spleen of mice by magnetic selection (Miltenyi) and activated with dynabeads CD3/CD28 at a 1:2 bead/cell ratio. Cells were cultured at a density of 10^6^ cell /ml in 12-well plates in RMPI culture medium supplemented with 10% FBS and antibiotics. One day later T cells were collected and resuspended in retroviral supernatant with 50 IU/mL rhIL-2 and 10 μg/mL protamine sulfate (Sigma), and spun at 2,000 g at 32°C for 90 min in 12 well plates. Infection was repeated at day 2. Lymphocytes were cultured with 100 IU rhIL-2 and subsequently split until day 5, when cells were used for functional analysis.

### Analysis of EGFR Expression and Signaling Pathway

Five days after retroviral (RV) transfection, transduced CD8^+^ T cells were harvested and GFP^+^ cells were sorted using a FACS Aria Sorter device (BD Biosciences). After 24 h, the expression of EGFR on the surface was measured by flow cytometry using EGF-APC conjugate (Life technologies). T cells were incubated with a 1:100 dilution of the ligand for 30 min at 37°C. After one wash with PBS, cells were analyzed by flow cytometry (FACS Calibur; BD Biosciences). For signaling studies, CD8^+^ T cells were lysed in RIPA buffer (Sigma) and homogenates were prepared and subjected to Western blot analysis as described previously (27) using anti-p-ERK1/2 (Thr202, Tyr204) and anti GAPDH as loading control (Cell Signaling; Beverly, MA).

### Functional Activity of Engineered Lymphocytes

Functional activity of transduced GFP CD8^+^ T cells (controls) or EGFR-GFP CD8^+^ T cells was measured by *in vitro* experiments. First, genetically modified OT-1 CD8^+^ T cells were stimulated with SIINFEKL peptide at a suboptimal (0.01 pg/ml) or optimal (10 μg/ml) concentration in the presence or absence of recombinant EGF (100 nM) for 24 h. The number of IFN-γ or TNF-α producing cells was analyzed by flow cytometry. Briefly, cells were incubated with Zombie NIR Fixable dye (Biolegend) and subsequently stained with fluorochrome-conjugated monoclonal antibodies (mAbs) against CD8 (53-6.7), CD4 (RM4-5) in the presence of purified anti-CD16/32 mAb. Cells were then fixed and permeabilized (eBiosciences) and then stained with anti-IFN-γ (XMG1.2), and anti-TNF-α (MP6-XT22) (BD Biosciences) mAbs. Samples were acquired on a FACSCanto-II cytometer (BD Biosciences). Data were analyzed using FlowJo software (TreeStar).

Also, genetically modified OT-1 T cells were cocultured with irradiated B16-OVA cells for 48 h, and proliferation rate and IFN-γ production were measured by ^3^H-timidine incorporation (0.5 μCi per well) and ELISA, respectively, as previously described (28).

Cytotoxic activity of modified OT1 cells was measured by a Real-time cytotoxicity assay (xCELLigence). In this assay, adhesion of cells to the gold microelectrodes impedes the flow of electric current between electrodes. The impedance value is plotted as a unit-less parameter called “Cell Index,” that increases as cells proliferate until cells approach 100% confluence. After the addition of B16.OVA cells to the wells, an initial phase of cell adhesion and spreading (0–6 h) is recorded before reaching a plateau phase (around 1 arbitrary CI). At this point, effector T cells are added and changes in cell index are recorded. The curve represents the mean Cell Index value from 3 wells ± SD. B16-OVA or B16F10 target cells were seeded in culture medium at a density of 20,000 cells per well (E-Plates 96 (Roche, Grenzach-Wyhlen, Germany). Cell attachment was monitored until the plateau phase was reached. Then, OT1 cells were added at different Effector:Tumor (E:T) cell ratios. Upon addition of effector cells, impedance measurements were monitored in real-time every 15 min during 24 h. An RTCA SP (Roche) instrument and the RTCA software Version 1.1 (Roche) were used to measure and analyze the data. All experiments were performed in duplicate.

Measurement of SIINFEKL specific IFN-γ producing cells after ACT. To evaluate the behavior of the modified CD8^+^ T cells *in vivo*, CD8 T cells obtained from C56BL/6 mice expressing the CD45.1 allele were modified genetically to express GFP or EGFR-GFP and injected into CD45.2 mice bearing B16-OVA tumors. Seven days after T cell infusion, T-cells producing IFN-γ were determined by ELISPOT (BD-Biosciences) following manufacturer's instructions. Briefly, splenocytes (7 × 10^5^/well) were stimulated with or without 1 μg/ml SIINFEKL peptide. After one day of culture the number of spot-forming cells was enumerated with an automated ELISPOT reader (CTL, Aalen, Germany).

### Adoptive Cell Transfer

C57BL/6 mice (6–12 weeks of age) were injected with 5 × 10^5^ B16-OVA melanoma cells or with 5 × 10^5^ PM-299L hepatoma cells by s.c route. Seven days later, mice were sublethally irradiated (total body irradiation) with 500 cGy and received 2–5*10^6^ CD8^+^ T cells from OT-I retrovirally transduced to express GFP or EGFR-GFP. Modified CD8+ T cells were purified by flow sorting of GFP^+^ cells before ACT experiments. The perpendicular diameters of the tumors were subsequently measured with a caliper. Mice were sacrificed when a tumor diameter reached a value >2 cm. The estimation of tumor volume was done using the modified ellipsoid formula 1/2(Length × Width^2^). For characterization experiments, B16-OVA tumor bearing mice (expressing CD45.2 allele) were treated with genetically modified CD8^+^ T cell (2*10^6^) from OTI × CD45.1 mice and 7 days later, mice were sacrificed to analyze by flow cytometry the presence of transferred cells into the tumors.

### Flow Cytometry

Excised tumors were digested with 400 U/mL collagenase D and 50 μg/mL DNase-I (Roche) for 20 min at 37°C. After washing with PBS, red cells were lysed by ACK buffer (Sigma). Spleens were mashed in PBS. For functional analyses, cells were stimulated with PMA (50 ng/ml)/Ionomycin (1 μg/ml) and GolgiStop and GolgiPlug (BD Biosciences). After 5 h, cells were incubated with Zombie NIR Fixable dye (Biolegend). Subsequently, they were stained with fluorochrome-conjugated mAbs against CD45.1 (A20), CD8 (XMG1.4), PD-1 (29F.1A12), LAG3 (C9B7W), and TIGIT (1G9) in the presence of purified anti-CD16/32 mAb. For intracellular staining, cells were fixed and permeabilized with the BD Fixation/Perm buffer (BD Biosciences) and then stained with anti-IFN-γ (XMG1.2) and with anti-KI67 (16A8) mAbs. Samples were acquired on a FACSCanto-II cytometer (BD Biosciences). Data were analyzed using FlowJo software (TreeStar).

### Statistical Analysis

Statistical analyses were performed using parametric (Student's *t* test and one-way ANOVA, and two-tailed paired *T*-test), and non-parametric (Mann-Whitney U and Kruskal-Wallis) tests. Kaplan-Meier survival curves were evaluated for statistical significance with the Log-rank Mantel-Cox test. For all tests a *p* value <0.05 was considered statistically significant. Descriptive data for continuous variables are reported as means ±SEM. GraphPad software was used for statistical analysis.

## Results

### EGFR and EGFR Ligand Expression in Murine Tumor Cell Lines and Solid Tumors

**Figure 1:**
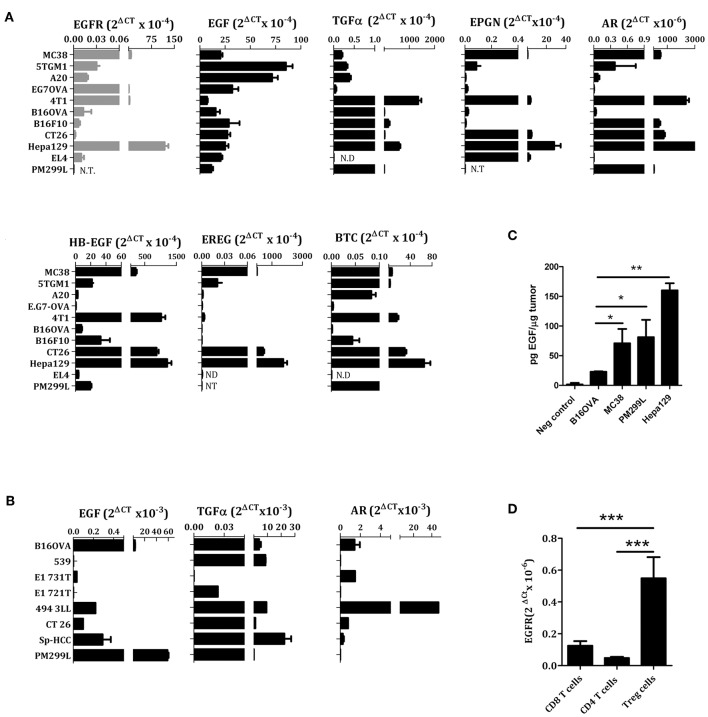
EGFR ligands and EGFR expression in different cell lines **(A)**, tumor biopsies **(B,C)**, and lymphocytes **(D)** analyzed by RT PCR. EL4, lymphoma; Hepa129 and PM299L, hepatocellular carcinoma; CT26, colon carcinoma; B16F10 and B16-OVA, melanoma; 4T1, breast cancer; EG7OVA, lymphoma; A20, reticulum cell sarcoma; 5TGM1, myeloma; MC38, colon carcinoma. **(C)** Amount of EGF in tumor cell extracts measured by ELISA, **(D)** EGFR expression in resting T cells. *p < 0.05; **p < 0.01; ***p < 0.005.

We examined the expression of EGFR and EGFR ligands using Real-time PCR in murine tumor cell lines and confirmed the broad expression of EGFR in tumors from different origin. Of note, we found a high expression of EGFR in Hepa 129, 4T1, EG7-OVA, and MC38, as compared to EL4, CT26, B16, A20, or 5TGM1 (Figure 1A). Regarding the EGFR ligands, we found that EGF was the predominant EGFR ligand in lymphoma, hepatocarcinoma, colon carcinoma, melanoma, breast cancer, myeloma and reticulum cell sarcoma cell lines (Figure 1A). For the remaining EGFR ligands, there was some heterogeneity of expression, both in cell lines and tumor biopsies obtained from mice (Figures 1A,B). The levels of EGF protein present into the tumor microenvironment were also measured by ELISA using tumor cell extracts obtained from mice bearing B16-OVA, MC38, PM299L, or Hepa129 cell line derived tumors. Interestingly, MC38, PM299L, and Hepa129 derived tumor extracts presented significantly higher EGF levels than B16-OVA melanoma extracts (Figure 1C).

### T Lymphocytes Can Be Transduced to Express Functional EGFR

It has been described that conventional human CD4 or CD8^+^ T lymphocytes do not express EGFR. However, Treg cells can express EGFR and benefit from EGFR ligands such as amphiregulin (22, 24). We have confirmed these results of EGFR expression using conventional murine CD4+ and CD8^+^ T cells as well as CD4^+^CD25^+^ Treg cells (Figure 1D).

**Figure 2:**
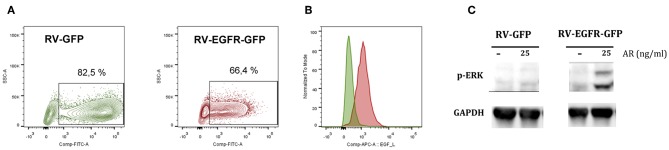
Genetically modified CD8 T cells express EGFR. **(A)** Percentage of CD8+ GFP+ cells after RV-GFP or RV_EGFR-GFP infection measured by flow cytometry. **(B)** EGFR expression on CD8 T cells transduced with RV-GFP and RV-EGFR-GFP cells, measured by flow cytometry using EGF-APC ligand. **(C)** Western blot analysis of phospho-ERK expression in CD8+ T cells transduced with RV-GFP and RV-EGFR-GFP. GAPDH was used as a loading control. Cells were left untreated or treated with 25 ng/ml Amphiregulin for 5 min. Results are representative of at least two experiments.

In order to modify genetically CD8^+^ T cells to respond to EGFR ligands, we generated a retrovirus for the simultaneous expression of EGFR and GFP using an IRES-containing bicistronic vector (RV-EGFR-GFP). As a control, we used a vector expressing only the GFP protein (RV-GFP). Infection of purified CD8^+^ T cells with RV-GFP vector was slightly more efficient (around 80% of transfection) than that achieved with RV-EGFR-GFP (around 65%). This difference is probably related to the size of EGFR gene. Indeed, although the cloning capacity of retroviral vectors is up to 10 kbps, the size of the transgene significantly influences its expression and viral titer (29). Also, the level of GFP expression was significantly lower in RV-EGFR-GFP than in RV-GFP as a result of the size of EGFR before the IRES sequence preceding GFP. Irrespective of these differences, in both cases (RV-EGFR-GFP and RV-GFP) the percentage of infected cells, measured by flow cytometry, was high (Figure 2A). Flow cytometry analysis using EGF labeled with APC showed that cells transduced with RV-EGFR-GFP expressed higher levels of EGFR than cells transduced with RV-GFP (Figure 2B).

EGFR triggering activates the ras/raf/MEK/MAPK pathway, comprising the activation of extracellular signal-regulated kinase (ERK) and JUN N-terminal kinase [reviewed in (30)]. In order to verify the adequate signaling of the transduced EGFR, we performed a western blot assay in cell extracts from CD8^+^ T cells transfected with RV-GFP control virus and RV-EGFR-GFP to measure MAPK/ERK pathway. A band of 42-44 kDa, corresponding to the putative molecular weight of p-ERK, was found in CD8^+^ T cells transduced with RV-EGFR-GFP but not with RV-GFP in response to the EGFR ligand amphiregulin (AR) (Figure 2C), suggesting the functionality of EGFR in lymphocytes sensing exogenous EGFR ligands. GAPDH protein was measured by specific WB and used to normalize the results.

### EGFR-Engineered CD8^+^ Lymphocytes Are Activated in Response to EGF

**Figure 3:**
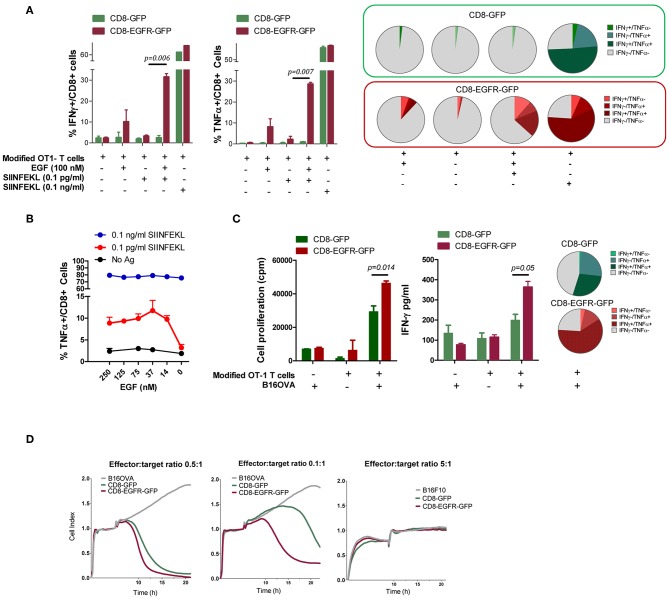
Effect of EGFR expression of T cell function. **(A)** Number of genetically modified OT-1 T cells producing IFN-γ and TNF-α after stimulation with SIINFEKL peptide in the presence/absence of EGF. Pie charts representing the percentages of T cells producing IFN-γ, TNF-α, or both cytokines. **(B)** Effect of different doses of EGF on CD8-EGFR-GFP T cell activation in response to high or low doses of SIINFEKL peptide. **(C)** T Cell proliferation and IFN-γ production of modified T cells in the presence or absence of irradiated B16-OVA cells, with a pie chart representing the percentages of T cells producing IFN-γ, TNF-α or both cytokines. **(D)** Capacity of modified CD8 T cells to recognize and lyse B16-OVA or B16F10 tumor cells, using the xCELLigence impedance-based system. Different T cell to tumor cell ratios were tested. Results are representative of at least two experiments.

T Cell Receptor (TCR) activation promotes a number of signaling cascades that ultimately determine cell fate through regulating cytokine production, cell survival, proliferation, and differentiation. There is a potential degree of crosstalk between TCR and EGFR induced signaling cascades. We speculated that EGFR-induced signaling might allow CD8^+^ T cells to respond better to simultaneous TCR stimulation. Therefore, CD8^+^ T cells from OT-1 mice were retroviraly modified to express GFP (CD8-GFP) or EGFR and GFP (CD8-EGFR-GFP) and stimulated with SIINFEKL peptide at two different concentrations (suboptimal: 0.1 pg/ml or optimal: 0.1 ng/ml) in the presence or absence of 100 nM EGF. The number of IFN-γ and TNF-α producing cells was analyzed by flow cytometry. The addition of EGF in the absence of SIINFEKL stimulation induced only a slight increase in the number of IFN-γ or TNF-α producing cells when CD8 T cells were modified to express EGFR (Figure 3A). When SIINFEKL peptide was added at a high concentration (0.1 ng/ml), no clear improvements were observed by the addition of EGF. However, when SIINFEKL concentration was suboptimal (0.1 pg/ml), a very significant improvement in the number of IFN-γ or TNF-α producing cells was observed in CD8 T cells transduced with EGFR. This increase was also evident when the number of double positive IFN-γ and TNF-α producing cells was analyzed (pie charts in Figure 3A). We also analyzed the effect of different doses of EGF on the activity of T cells exposed to high (0.1 ng/ml) or low (0.1 pg/ml) doses of SIINFEKL peptide. As it is shown in Figure 3B, EGFR transduced OT-1 T cells did not respond efficiently to low doses (0.1 pg/ml) of SIINFEKL. However, addition of as low as 14 nM of EGF allowed the production of TNF-α in response to this peptide concentration. On the other hand, when SIINFEKL was added at high concentration (0.1 ng/ml) almost 80% of the EGFR transduced OT-1 T cells produced TNF-α and no beneficial effect was observed by the addition of EGF to the co-cultures. These results may suggest that EGFR signaling may synergize with TCR signaling when the latter is suboptimal.

Modified OT-1 T cells were also stimulated by co-culture with irradiated B16-OVA tumor cells and IFN-γ production and T cell proliferation was measured after 48 or 72 h, respectively. Interestingly, CD8-EGFR-GFP proliferate significantly better in response to B16-OVA cells and produce higher levels of IFN-γ than CD8-GFP transduced cells (Figure 3C). These differences were also observed in the number of double positive IFN-γ and TNF-α producing cells measured by flow cytometry, suggesting that EGFR signaling is favoring the activation of a polyfunctional immune response.

To evaluate a potential benefit that EGFR ligands produced by tumor cells might provide to modified T cells for tumor recognition, we examined the capacity of EGFR-transduced OT-1 CD8^+^ T cells to recognize and lyse tumor cells expressing ovalbumin. Using the xCELLigence impedance-based system we found that both CD8-GFP and CD8-EGFR-GFP T cells were able to recognize and kill B16-OVA cells in a similar way when the effector to target ratio was relatively high (0.5 effector T cells per 1 tumor cell) (Figure 3D, first graph). However, when the E:T ratio was five times lower (0.1 effector T cells per 1 tumor cell), CD8-EGFR-GFP T cells were clearly more efficient killing B16-OVA cells with respect to CD8-GFP cells (Figure 3D, second graph). This antitumor response was antigen-specific since neither CD8-EGFR-GFP or CD8-GFP were able to kill B16F10 tumor cells even at a high E:T ratio (5:1) (Figure 3D, third graph).

### EGFR-Expressing CD8^+^ T Cells Exerted Stronger Antitumoral Activity *in vivo*

**Figure 4:**
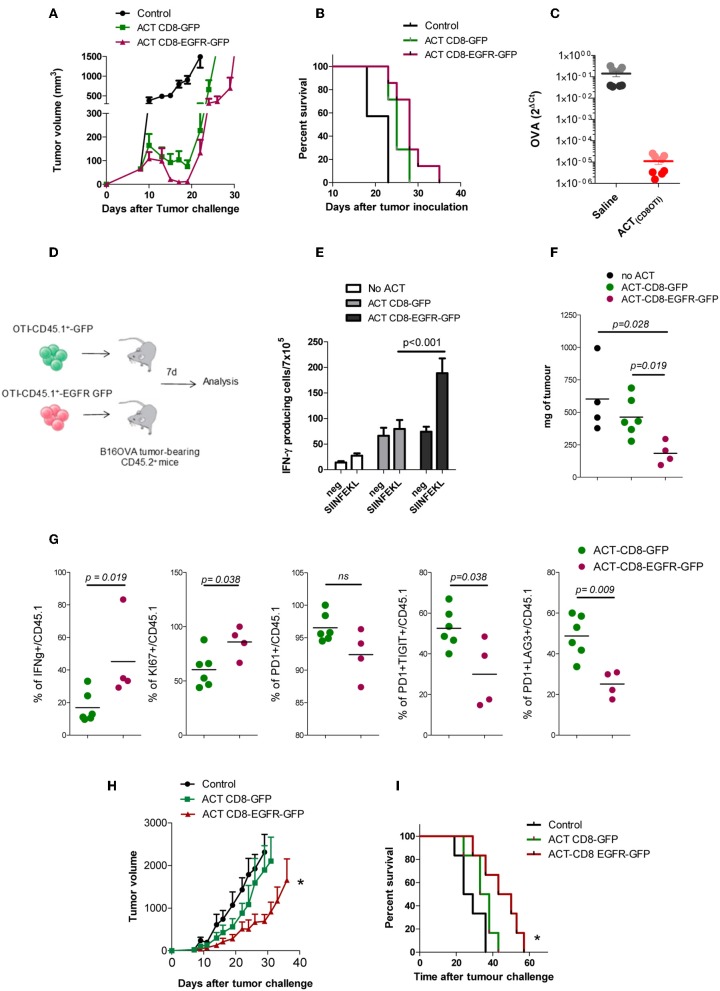
*In vivo* effect of EGFR expressing CD8+ T cells *in vivo*. **(A)** B16-OVA tumor growth after adoptive transfer of OT-1 modified T cells. **(B)** Kaplan–Meier plots of survival of mice bearing B16-OVA tumors. **(C)** Levels of OVA expression on tumor tissue isolated from B16.OVA bearing tumor mice 10 days after ACT with OT1-T cell or with saline (two mice per group, 4 experimental replicates per sample) **(D)** Schematic of experimental design. **(E)** ELISPOT analysis of IFN-γ producing cells in response to SIINFEKL peptide in splenocytes, 7 days after adoptive transfer of genetically modified CD8 OT1 T cells. **(F)** Tumor weight (in mg) 7 days after adoptive T cell therapy. **(G)** Functional and phenotypic analysis of tumor infiltrating CD45.1 T cells 7 days after adoptive T cell transfer. **(H)** PM299L tumor growth and **(I)** Kaplan Meyer survival plots after adoptive transfer of OT-1 modified T cells. Results are representative of at least two experiments. *p < 0.05.

We wondered if EGFR-engineered CD8^+^ lymphocytes would exhibit an enhanced therapeutic effect *in vivo* in tumor-bearing mice. Thus, C57BL/6 mice were injected with B16-OVA cells and at day 7, when tumors reached 5 mm in diameter, 5 × 10^6^ OT1 T cells modified with RV-EGFR-GFP or RV-GFP were injected i.v. into the mice. Although there was a trend to a higher reduction of tumor size immediately after the adoptive transfer of CD8-EGFR-GFP cells, compared to that found with CD8-GFP cells, this therapeutic effect was not statistically significant and it was lost soon after T cell transfer (Figures 4A,B). This lack of efficacy might be associated to the loss of OVA antigen expression in B16-OVA cells due to immune pressure, as it has been described previously (31). In a parallel experiment, we evaluated the level of expression of OVA antigen in tumors 10 days after adoptive transfer of 5 × 10^6^ OT1 T cells. As it is shown in Figure 4C, OVA expression measured by RT-PCR was dramatically reduced (around 4 logs) in mice treated with OT-I T cells with respect to mice treated with saline (Figure 4C). These data suggest that immune pressure on the target B16.OVA tumor cells *in vivo* promoted the appearance of tumor cells characterized by loss target OVA antigen expression as it has been described previously (32–34).

To evaluate the behavior of the modified CD8^+^ T cells in vivo, we repeated the experiment of adoptive transfer but using CD8 T cells obtained from C56BL/6 mice expressing the CD45.1 allele. Thus, CD45.1 T cells were modified genetically to express GFP or EGFR-GFP and injected into CD45.2 mice bearing B16-OVA tumors (Figure 4D). Seven days after T cell infusion we analyzed by ELISPOT the capacity of T cells to produce IFN-γ in response to 1 μg/ml SIINFEKL stimulation. It was found that splenocytes from mice transferred with CD8-EGFR-GFP cells had a higher number of SIINFEKL-specific IFN-γ producing cells (Figure 4E). The size of the tumor at day 7 after T cell transfer was also significantly lower in mice transferred with CD8-EGFR-GFP cells (Figure 4F). The CD45.1 marker allowed us to analyse by flow cytometry the behavior of transferred T cells infiltrating the tumor. Although no significant changes in the number of CD8^+^ T cells infiltrating the tumor were observed (data not shown), we found a significantly higher percentage of CD45.1+ IFN-γ producing cells into the tumor of mice treated with CD8-EGFR-GFP as compared to that found in mice treated with control CD8-GFP T cells. The % of proliferating cells (KI67+ cells) was significantly higher in tumor infiltrating CD8-EGFR-GFP T cells and they appeared to be less dysfunctional. Indeed, a significantly lower percentage of PD1^+^TIGIT^+^ or PD1^+^LAG3^+^ CD8^+^ T cells was observed in tumors from mice injected with CD8-EGFR-GFP cells (Figure 4G) with respect to that found in tumors from mice transferred with CD8-GFP T cells.

We then studied if EGFR-engineered CD8^+^ lymphocytes would have a better therapeutic effect on mice bearing PM299L tumors, a tumor cell line derived from fresh hepatic tumors induced in C57BL/6 mice as previously described (35). PM299L tumors express SIINFEKL and grow very efficiently when they are injected s.c. in C57BL/6 mice. In this model, we found a significant delay in tumor growth (Figures 4H,I) and a statistically longer overall survival in mice adoptively transferred with CD8-EGFR-GFP T cells as compared to untreated mice or to mice receiving CD8-GFP T cells.

## Discussion

The efficacy of adoptive T cell therapy is highly compromised by the tumor microenvironment. It has been shown in preclinical models that, just 24 h after tumor-specific CD8^+^ T cell transfer, these cells become hypofunctional and express inhibitory receptor proteins that converge in cell cycle inhibition (36). Persistence and survival of the genetically modified T cells is considered a key factor contributing to the efficacy of ACT (37). We speculated that if adoptive transferred T cells could benefit from cytokines presented in the tumor microenvironment they could exert better anti-tumor responses. Among the potential alternatives we focussed on EGFR ligands.

EGFR utilizes a network of downstream signaling events leading to cell growth, proliferation, survival, angiogenesis, migration, and metastasis of many cancers. Its signaling can provide substantial advantage in tumor cell survival. We first analyzed the expression of EGFR and their ligands in a panel of tumor cell lines as well as tumor biopsies obtained from mice previously inoculated with the tumor cell lines. These experiments demonstrated that EGFR and its ligands are broadly expressed in murine tumors as occurred in humans (38, 39).

EGFR is ubiquitously expressed in normal tissues albeit at a much lower level than on tumors. But, in general, is thought to be absent in the hematopoietic cell linage, with the exception of some monocytes upon activation (20). Recently, several reports have shown that Treg cells express EGFR and enhance their immunosuppressive activity after stimulation with the EGFR ligand AR (22–24). In contrast, effectors T cells poorly express EGFR [(22), and confirmed in the present study], so that they do not benefit from the pro-proliferative and pro-survival signals provided by these ligands in the tumor microenvironment. In this scenario, the purpose of the current study was to modify genetically anti-tumor CD8^+^ cells to express EGFR to take advantage of the proliferative effect that EGFR ligands produced in the tumor microenvironment.

We have found that retrovirus RV-EGFR-GFP can efficiently modify CD8^+^ T cells to express EGFR and activate the MAPK/ERK pathway in response to EGFR ligands. It was also shown that T cells modified with RV-EGFR-GFP were able to respond to EGF improving their proliferation rate as well as the IFN-γ and TNF-α production capacity in response to a suboptimal TCR stimulation as compared to cells modified with the control RV-GFP. This improvement was not so clear when cells were treated with saturating concentration of antigen, probably because the induction of an optimal activation may make difficult to see any potential improvement induced by EGF. However, the TCR stimulation induced by tumors *in vivo* is by far, lower than the *in vitro* stimulation with saturating concentrations of SIINFEKL peptide used in our *in vitro* assays. Indeed, it has been reported that T cell stimulation in the tumor microenvironment is, in general, suboptimal (40). Similarly, CD8-EGFR GFP cells were able to kill more efficiently OVA expressing tumors than CD8-GFP cells when the E:T ratio was low. Thus, we believe that under the patho-physiological conditions present in many types of cancers (low E:T ratio, low antigen concentration, impaired antigen presentation and high immunosuppressive microenvironment), the presence of EGFR ligands in tumors might improve the effector functions of EGFR modified T cells.

ErbB ligands and receptors activate a complex signaling network including the ras/raf/MEK/ MAPK and the PI3K pathways and the activation of various transcription factors such as c-fos, c-Jun, c-myc, STAT, NF-kB, zinc finger transcription factor and Ets family members (41). Our i*n vitro* experiments using antigen specific CD8^+^ T cells suggest that expression of EGFR on T cells might give a functional advantage in a tumor microenvironment containing EGFR ligands. EGFR-expressing effector T cells proliferate better and produce more IFN-γ and TNF-α in the presence of EGFR ligands produced by tumor cells *in vitro* and exerted stronger antitumor response delaying tumor growth *in vivo*. However, our results *in vivo* are not optimal, and the tumors still escape the action of the transferred T cells.

EGFR modified T cells could benefit from EGF ligands if their concentration into the tumors falls into the range of concentrations leading to augmented T cell activity. Although several works have estimated the concentration of EGFR ligands in human body fluids (42, 43) there are not clear data about their concentration in tumor tissues. We have quantified by ELISA the EGF levels in different tumors types isolated from mice previously inoculated with tumor cell lines. EGF levels ranges between 20 pg/μg tumor (in B16.OVA) to 160 pg/μg tumor (in Hepa129). A rough estimation using these data might suggest the presence of a tumor concentration of EGF 1 log below the concentration used in our *in vitro* assays. However, it is difficult to extrapolate these results to the *in vivo* setting since EGFR might respond not only to EGF but also to TGF-α, AR, EPGN, BTC, HB-EGF and EREG, which are also increased in tumor tissues (9–15). A recent study by Pinilla et al. has estimated a range of EGFR ligand concentrations in tumors as low as ~0.2 and ~0.6 ng/ml (~34 and 100 pM) (44). They have also shown that the levels of phosphorylation and ubiquitylation of EGFR in tumors *in vivo* closely resemble the levels observed in the same tumor cells treated with 20–200 pM EGF *in vitro*. The authors conclude that a small pool of active EGFRs is sufficient to activate efficiently EGFR signaling. Considering these results, we might guess that the retrovirus-transduced CD8 T cells to express EGFR could benefit from EGFR ligand concentrations found in the tumor microenvironment.

Tumors can escape from T cell attack in a variety of ways. They can promote T cell exhaustion, the expansion of suppressive cells, or become resistant to CTL killing by downregulating MHC-I expression or by losing the target antigen. In agreement with previous reports (31–33) we observed that *in vivo* ACT rapidly promoted the emergence of tumor cells negative for OVA antigen suggesting that potent immunotherapies can actively promote tumor evolution. Loss of tumor antigen expression in our models might have an impact in the anti-tumor activity of EGFR expressing T cells. But, in addition to this phenomenon, there are other barriers in the tumor microenvironment that need to be overcome for a better antitumor efficacy of adoptive cell therapy.

**Figure 5:**
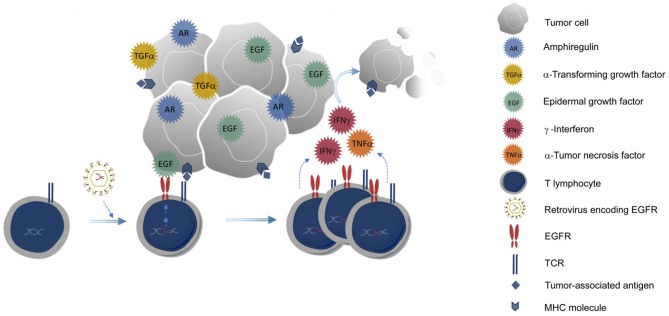
Genetic modification of CD8+ T cells to express EGFR. T cells expressing EGFR could benefit from EGFR ligands produced by the tumor, proliferate better, and exert their anti-tumor activities more efficiently into the tumor microenvironment.

The potential effect of EGFR and its ligands on the immune system is still poorly understood. Indeed, recent reports suggest that EGFR expression by leukocytes in the functioning of the immune system might be underestimated (26, 45). Cancer patients treated with EGFR antagonists suffer not only from a wide range of side effects caused by loss of EGFR function in epithelial cells but also become more susceptible to infections (46) probably due to effects on the immune system (47). Our results suggest that the genetic modification of CD8^+^ T cells to express EGFR might endow them with a proliferative advantage to fight against tumors expressing EGFR ligands (Figure 5). EGFR expressing T cells could also compete for EGFR ligands with tumor cells and Treg cells as has been described for soluble EGFR (48), and thus might also have a positive effect on tumor control. Although further experiments are needed to elucidate the potential crosstalk between different signaling cascades and the final outcome of the modified T cells into the tumor microenvironment, our results suggest a potential benefit of EGFR expression on T cells that should be considered in immunotherapeutic strategies based on adoptive transfer with anti-tumor T cells.

## Data Availability Statement

All datasets generated for this study are included in the article/supplementary material.

## Ethics Statement

Animals were housed in the accredited animal facility of the Center for Applied Medical Research, CIMA, and animal experimentations were performed after approval by and according to the guidelines of the Animal Experimentation Ethical Committee of the University of Navarra and The Local Authority for the use of laboratory animals (protocol R-131-16GN).

## Author Contributions

TL, SC, and NC performed most of the experiments. JL and NC conceived the study and participated in the design and interpretation of results. CV, MG, MR, PSarr, PSaro, AL-C, CM, AL, SH-S, and JL participated in the experiments. JL wrote the first draft and NC, TL, SC, SH-S, and PSaro participated in the manuscript writing. All authors read and approved the final manuscript.

### Conflict of Interest

The authors declare that the research was conducted in the absence of any commercial or financial relationships that could be construed as a potential conflict of interest. The reviewer CA declared a shared affiliation, with no collaboration, with several of the authors JL, TL, SC, CV, MG, CM, AL-C, SH-S, PSaro, PSarr, and NC to the handling editor at the time of the review.
